# Cecal Bascule in a COVID-19 Positive Patient: Case Report

**DOI:** 10.1055/s-0042-1743527

**Published:** 2022-03-03

**Authors:** Arthur Curmi, Robert Cuschieri

**Affiliations:** 1 Department of Surgery, Mater Dei Hospital, Triq Dun Karm, Msida MSD, Malta

**Keywords:** volvulus, bascule, cecum, obstruction, anastomotic leak, case report

## Abstract

Cecal volvulus is an uncommon cause of acute intestinal obstruction accounting for around 10% of intestinal volvuli. There are three main variants of cecal volvuli including the axial, loop, and bascule types. Diagnosis is confirmed via a computed tomography scan and surgery is the mainstay treatment due to a high risk of morbidity and mortality. Here we report a rare presentation of cecal volvulus in a COVID-19 positive patient that was complicated by an anastomotic leak.

A volvulus is when a loop of bowel twists on its mesentery that supports it. 1 It most commonly occurs in the sigmoid colon (80%), followed by the cecum (15%), transverse colon (3%), and splenic flexure (2%). 2 Cecal volvulus is, therefore, an uncommon cause of acute intestinal obstruction and accounts for around 10% of intestinal volvuli. 3 It is more common in women than in men and usually presents in the third decade of life compared with sigmoid volvulus which normally occurs in the elderly population. 4

Cecal bascule is a rare form of volvulus that is characterized by upward and anterior folding of the cecum. 5 Etiology is a multifactorial process and predisposing factors include failure of peritoneal fixation during embryonic development allowing for a free and mobile cecum, increased age, long-standing constipation, previous abdominal surgery, prolonged immobility, high-fiber diet, and use of psychotropic drugs. 5 In the presence of a competent ileocecal valve, cecal bascule may progress to closed loop obstruction. 5 Treatment usually involves urgent surgical intervention as delay may lead to bowel necrosis and perforation. 6 We present the first reported case of cecal bascule in a COVID-19 positive patient.

## Case Report

**Fig. 1:**
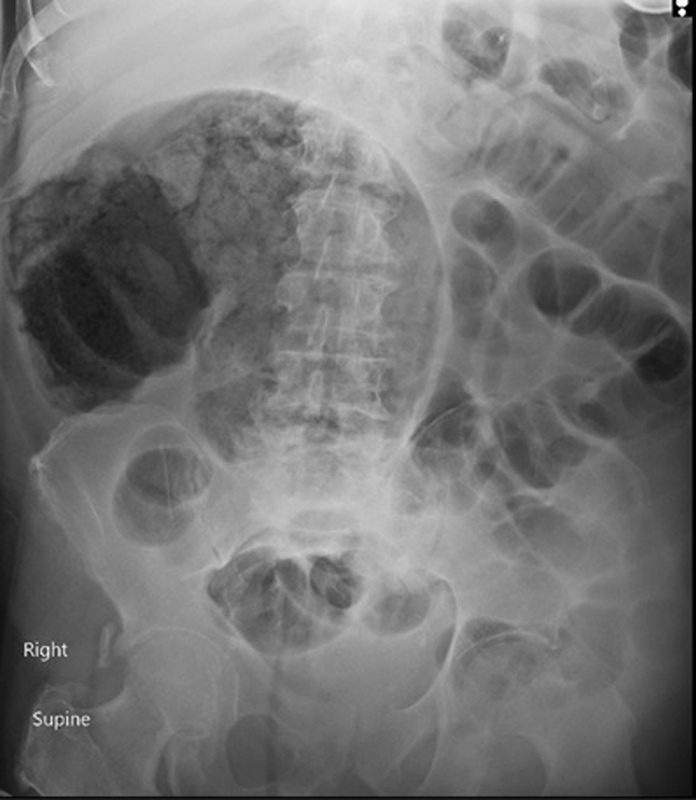
Abdominal X-ray showing dilated large bowel loop in the right side of the abdomen.

**Fig. 2:**
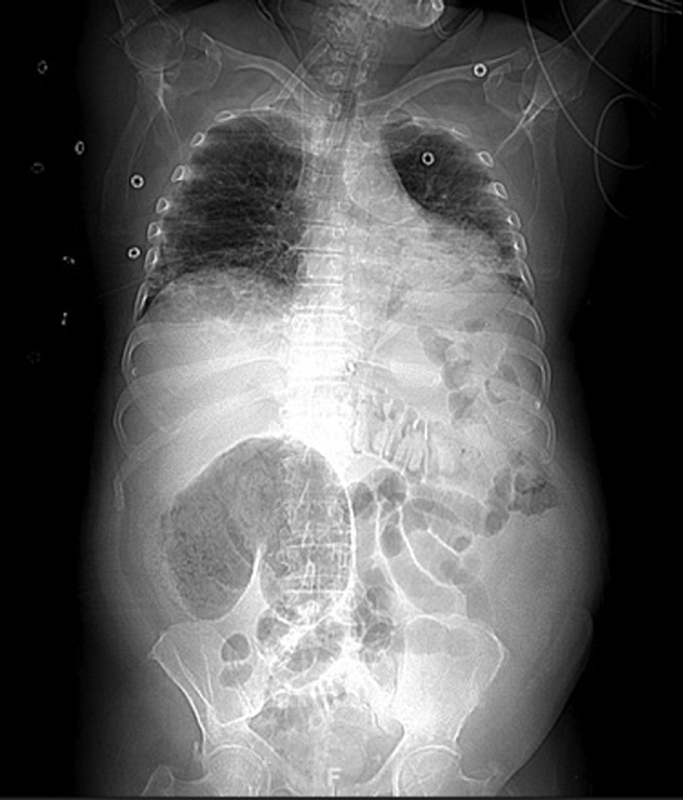
Scout view of CT abdomen/pelvis showing a distended cecum lying anterior and medial to its normal position.

**Fig. 3:**
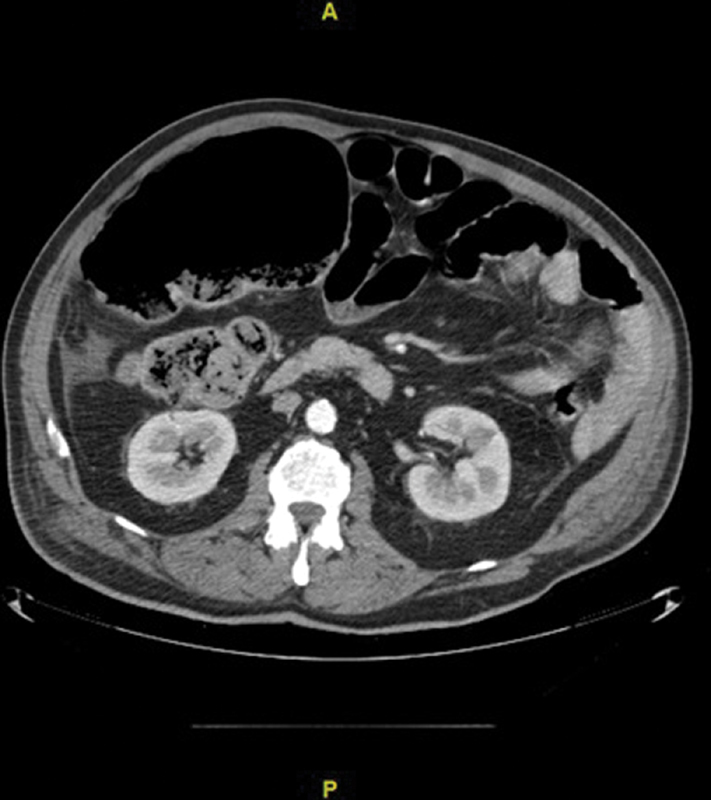
Abnormally distended cecum on axial view of CT abdomen/pelvis.

A 68-year-old male was directly admitted to the intensive therapy unit in view of COVID-19 pneumonia on CT thorax. His past medical history was remarkable for congestive heart failure, hypertension, ischemic heart disease, and diabetes mellitus. He was an ex-smoker of one packet daily and previously independent in his activities of daily living with a good exercise tolerance. While he was in ITU he was on mechanical ventilation and was receiving enteral nutrition via a nasogastric tube. On day 12 of his ITU stay, he was noted to have increased abdominal distension, generalized abdominal tenderness, and bowels were not opened for the previous few days. His bloods were as follows: WBC: 23.43 × 109/L, hemoglobin: 11.2 g/dL, CRP: 32.3 mg/L, EGFR: 91. A surgical consult was sought, and an abdominal X-ray showed dilated large bowel loops in the right side of the abdomen ( Fig. 1 ). Subsequent CT scanning of the abdomen showed an abnormally distended cecum (measured up to 10 cm on axial plane) which lied anterior and medial to its normal position ( Figs. 2 and 3 ). There was some surrounding fat stranding and a small amount of free fluid. The ascending and transverse colons were not significantly distended and were occupied by feces while the rest of the large bowel was collapsed. The impression was of cecal bascule. He underwent an emergency laparotomy which confirmed a cecal volvulus with severely distended cecum and extensive serosal tear. There was no perforation or fecal spillage. Since the patient was hemodynamically stable with no signs of sepsis and not receiving any inotropes or vasopressors at the time of surgery, it was decided to proceed with a right hemicolectomy and primary ileocolic anastomosis. A drain was inserted in the right paracolic gutter. This was later removed after the patient had opened his bowels. On day 11 postoperatively, he was febrile, had increased nasogastric tube aspirate, reduced urine output, and rising inflammatory markers. A second CT abdomen/pelvis was performed which revealed a leak at the site of the anastomosis. A further resection was performed at laparotomy and both bowel ends were externalized as an ileostomy and a mucous fistula, respectively. Following the second laparotomy, he developed septic shock, multiorgan failure syndrome and passed away 1 day later.

## Discussion

There are three main presentations of cecal volvulus: axial, loop, and bascule. Type 1 (axial type) is the clockwise or counterclockwise axial twisting (180–360 degrees) of the cecum along its long axis with the cecum remaining in the right lower quadrant of the abdomen. 5 Type 2 (loop type) usually involves counterclockwise torsion of both cecum and terminal ileum with the cecum displaced to an abnormal position, typically the left upper quadrant. 5 Our case had type 3 (bascule type) cecal volvulus, characterized by anterior upward folding of the cecum but no axial twisting that is seen with the more common type of volvuli. 5 Our patient most likely developed this condition secondary to his history of constipation while on enteral feeds in ITU. The axial and loop type account for around 80% of all cecal volvuli with the cecal bascule accounting for the remaining 20%. 3 Blood supply is rarely compromised in cecal bascule, as the mesentery is not frequently twisted compared with other types of volvuli.

Patients usually present with signs of intestinal obstruction including severe abdominal pain, abdominal distension, constipation, and vomiting. On examination there is tympanitic abdomen to percussion and bowels sounds are absent on auscultation. In the context of gangrene or perforation, the cecal volvulus presents with an acute abdomen, signs of peritonism and septic shock. 7 The clinical presentation may vary according to the length of bowel involved and the extent and duration of the twist, hence, the symptoms and clinical signs are often not specific enough to ensure an early diagnosis. 3

Laboratory investigations are neither sensitive nor specific for the diagnosis of cecal volvuli. Abdominal radiography may show small bowel and cecal dilatation, air fluid levels anywhere in the abdomen depending on the volvulus type, and absence of gas in the distal colon. These features, however, are non-specific in nature and 30% of the affected individuals do not show any of these radiographic findings making diagnosis difficult. 8 The associated presence of an appendix distended with air, may help in eliciting a possible diagnosis of cecal volvulus. 9

Barium enema was traditionally used to confirm the diagnosis of cecal volvulus but in view of the longer duration of the investigation and possibility of barium extravasation, it has been largely replaced by computed tomography as the imaging investigation of choice. 10 Common findings on CT include the coffee bean, bird beak, and whirl signs. 11 The coffee bean is characterized by juxtaposition of the medial walls of the dilated cecum creating the central cleft of the coffee bean while the lateral walls of the distended bowel form the outer parts of the bean. 12 Bird beak refers to the gradually tapering loops of bowel which then end at the site of obstruction. 13 The whirl sign is more specific for volvulus and consists of spiraled loops of collapsed cecum. 13 Besides distinguishing between different types of cecal volvulus, a CT also helps in the diagnosis of potential complications including pneumoperitoneum, pneumatosis intestinalis, circumferential wall thickening, and increased mesenteric fat density. 13

Surgical intervention is the mainstay treatment for all types of cecal volvulus. Immediate surgical reduction of the twisted bowel prevents necrosis decreasing both morbidity and mortality. 14 In cases of gangrene and perforation, resection should be performed either via open or laparoscopic approaches. 3 The affected bowel should also be removed in cases of gross dilatation with a thin-walled cecum 15 as was with our case. After resection, reconstruction as primary anastomosis or ileostomy with a mucous fistula depends on the physical status of the patient prior to surgery as well as intraoperative findings. 16 Patients who are hemodynamically stable with no bowel compromise should undergo a right hemicolectomy with primary ileocolic anastomosis. 16 In patient with hemodynamic instability and compromised bowel the surgeon should opt for an ileostomy following bowel resection. 16 The ileostomy could be reversed at a later operation. In addition to resection, cecopexy should be performed to fix the remnant right colon to the posterior abdominal wall and reduce the risk of future volvuli. 16 In our case, a right hemicolectomy with primary ileocolic anastomosis was performed since the patient was hemodynamically stable and not on any inotropes at the time of surgery. Also, there was no bowel compromise or any fecal spillage at laparotomy, hence, we believe that this was the right treatment option for the patient at that time.

A high mortality rate has been reported following surgical intervention for cecal volvulus. 11 Postoperative complications include cardiac and pulmonary events as well as other surgical complications such as paralytic ileus, wound infection, and anastomotic leak. Our patient had an anastomotic leak on day 11 post right hemicolectomy with primary ileocolic anastomosis while still COVID-19 positive. Following further bowel resection, our patient had an ileostomy created at a second operation. We now argue whether we should have considered an ileostomy at first operation given the high morbidity and mortality associated with COVID-19 patients. A recent study suggested poor outcomes for patients undergoing surgery during the COVID-19 pandemic with one in four patients dying within 30 days after operation. 17

## Conclusion

Cecal bascule is a rare form of cecal volvulus, which itself is a very rare surgical condition. This is also the first reported case of a cecal bascule in a COVID-19 positive patient. Prompt recognition and urgent surgical intervention is essential to reduce mortality rates and improve long-term patient outcomes. The question is whether a stoma needs to be considered at the initial operation in this new group of patients irrespective of stable patient and bowel at surgery, given the poor prognosis associated with COVID-19.
